# Do we know others' visual liking?

**DOI:** 10.1068/i0661

**Published:** 2014-11-20

**Authors:** Ryosuke Niimi, Katsumi Watanabe

**Affiliations:** Department of Psychology, Graduate School of Humanities and Sociology, The University of Tokyo, Tokyo, Japan; e-mail: niimi@L.u-tokyo.ac.jp; Research Centre for Advanced Science and Technology, The University of Tokyo, Tokyo, Japan; e-mail: kw@fennel.rcast.u-tokyo.ac.jp

**Keywords:** object perception, preference, aesthetics, false consensus effect, gender difference

## Abstract

Although personal liking varies considerably, there is a general trend of liking shared by many people (public favour). Visual liking in particular may be largely shared by people, as it is strongly influenced by relatively low-level perceptual factors. If so, it is likely that people have correct knowledge of public favour. We examined the human ability to predict public favour. In three experiments, participants rated the subjective likability of various visual objects (e.g. car, chair), and predicted the mean liking rating by other participants. Irrespective of the object's category, the correlation between individual prediction and actual mean liking of others (prediction validity) was not higher than the correlation between the predictor's own liking and the mean liking of others. Further, individual prediction correlated more with the predictor's own liking than it was with others' liking. Namely, predictions were biased towards the predictor's subjective liking (a variation of the false consensus effect). The results suggest that humans do not have (or cannot access) correct knowledge of public favour. It was suggested that increasing the number of predictors is the appropriate strategy for making a good prediction of public favour.

## 1 Introduction

Liking—that is, affective attitude towards objects, people, and events—is difficult to understand. Humans like some and dislike others, but underlying rules and mechanisms of liking are obscure in most cases—there is no accounting for tastes. Nevertheless, scientists, including psychologists, have tried to explain liking, as it frequently influences human behaviour, ranging from daily shopping to elections.

Liking is a complex phenomenon influenced by multiple perceptual, psychological and social factors, including perceptual regularity, personality, culture and so forth. For example, the popularity of photographs—as measured by the number of views in an online community (Flickr)—is related to a number of variables, ranging from image features (colour, object) to social features of the individual who uploaded the photograph (Khosla, Sarma, & Hamid, 2014). Recent studies have demonstrated, however, that visual liking is determined very fast and even unconsciously. For instance, subjective likability of faces and common objects is reliably determined within 100 ms of stimulus presentation (Niimi & Watanabe, 2012; Willis & Todorov, 2006). Results of neurophysiological experiments have also suggested that the human brain automatically encodes subjective values of visual objects. Activities of reward-related brain regions such as the orbitofrontal cortex (OFC), cingulate cortex and striatum correlate with aesthetic/economic values (Ishizu & Zeki, 2011; Kawabata & Zeki, 2004; Padoa-Schioppa & Assad, 2006; Plassmann, O'Doherty, & Rangel, 2007; Yue, Vessel, & Biederman, 2007), and some of those regions encode subjective values even when the observer is not engaged in a value-related task (Kim, Adolphs, O'Doherty, & Shimojo, 2007; Lebreton, Jorge, Michel, Thirion, & Pessiglione, 2009; Tusche, Bode, & Haynes, 2010). Further, relatively low-level visual features, such as image contrast, contour roundedness, symmetry and complexity influence visual likability (Bar & Neta, 2006; Jacobsen, Shubotz, Höfel, & von Cramon, 2006; Tinio, Leder, & Strasser, 2011). Perceptual fluency also affects likability (Reber, Winkielman, & Schwarz, 1998). For face attractiveness, averageness and symmetry are suggested to be the determinants (Thornhill & Gangestad, 1999; but see DeBruine, Jones, Unger, Little, & Feinberg, 2007; Halberstadt & Winkielman, 2014). Thus, it is apparent that visual liking is influenced by not only high-level social factors but also lower, perceptual factors. Models of liking judgment should contain multiple processing stages (Leder, Belke, Oeberst, & Augustin, 2004; Lindel & Mueller, 2011).

If a considerable part of liking judgment is determined by perceptual factors, it is not surprising that there is a general trend of liking shared among many people; some are liked by many people and become popular, while others are not. It has been reported that there is a considerable consensus among people for picture preference (Vessel & Rubin, 2010) and face attractiveness ratings (Wood & Brumbaugh, 2009). Therefore, liking can be described as having two components, namely, public favour and idiosyncratic liking (e.g. Hönekopp, 2006). The former may be more likely to be determined by perceptual factors. It seems harder to identify the origins of idiosyncratic liking, but familiarity may be proposed to play a role (Reis, Maniaci, Caprariello, Eastwick, & Finkel, 2011; Zajonc, 1968).

Knowing the common, average liking of others (public favour) is critical for human social behaviour. For instance, if we do not know a person beforehand, we will choose a popular product as a present. In this study, we questioned whether thinking and predicting public favour of others is actually helpful.

Social psychology tells us that it would be difficult because people often misperceive social consensus. The false consensus effect (FCE) is the phenomenon that people overestimate the consensus between one's own and others' opinions (Krueger, 1998; Marks & Miller, 1987; Ross, Greene, & House, 1977). For instance, a person is asked to express his/her opinion about a given issue (e.g. vote for/against a new nuclear plant) and to estimate percentage consensus, that is, the percentage of peers who share the opinion. On average, the estimated consensus is higher than the actual consensus. The effect is known to occur with liking and disliking judgments (e.g. Gershoff, Mukherjee, & Mukhopadhyay, 2008; Gilovich, 1990; but see Nisbett & Kunda, 1985). However, under some conditions, FCE is not observed and even presents as the opposite phenomenon (false uniqueness effect; Bosveld, Koomen, van der Pligt, & Plaisier, 1995; Suls & Wan, 1987).

Most studies of FCE used materials that were presented as written word stimuli rather than visual stimuli from various categories (e.g. film, music genre, actor, sundae; Gershoff et al., 2008 used movie posters as stimuli, though the experiment did not focus on visual liking). If people explicitly know the public favour of visual stimuli and discriminate it from their own liking, predicting others' liking would correlate better than simply stating one's own liking. To address the issue, we conducted three experiments in which participants reported their own liking (rating task) and predicted average others' liking (prediction task) for visually presented common objects.

## 2 Experiment 1

### 2.1 Method

#### 2.1.1 Stimuli

**Figure 1.**
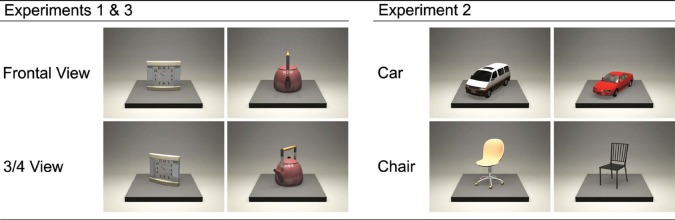
Examples of the stimuli. Experiments 1 and 3 adopted identical sets of stimuli, which included various object categories. The objects were shown in either frontal view or 3/4 view. Experiment 2 examined two sets of single-category objects (cars and chairs; 3/4 view only). See Additional Material for the entire list of objects.

The stimuli were computer-generated colour images of 38 common objects (32 for experimental trials, 6 for practice trials; see Additional Material at http://i-perception.perceptionweb.com/journal/I/volume/5/article/i0661). They were created by 3D computer graphic software (Shade 9, e-frontier Inc., Tokyo), and had been used as stimuli in another study (Niimi & Watanabe, 2012). We included various object categories (e.g. furniture, vehicles and kitchenware). The objects were placed on a square stage and oriented to the camera (frontal view) or 30º rotated (3/4 view), resulting in 76 stimulus images (64 for experimental and 12 for practice trials; see Figure 1 for examples).

#### 2.1.2 Procedure

The experiment consisted of a rating task and a predicting task. The apparatus, stimuli and procedure—except the instructions—were identical between the two tasks. In the rating task, participants were instructed to rate the visual likability of the object, that is, “how good is the object's appearance?” Participants were also told that the task was to rate the visual likability of the object, not necessity/wanting. In the prediction task, the experimenter first explained the procedure of the rating task. As a fictional cover story, the participants were told that 20 individuals (11 males, 9 females) living in Tokyo had performed the rating task and they were 21.4 years of age on average, ranging from 18 to 36 years. They were then asked to predict the result, namely, the average of the likability ratings made by the 20 fictional participants. The age and gender data in the cover story were obtained from a group of participants in another experiment not reported in this paper, as it was a good estimation of age/gender of participants recruited by the authors' laboratory.

The rating/prediction was reported through a response box on which seven buttons were horizontally aligned. The buttons were marked by number (1–7), simulating a 7-point Likert scale ranging from 1 (“very unlikable”) to 7 (“very likable”). Button 4 indicated a neutral response (“neither”).

In each trial, a stimulus image was shown at the centre of a computer screen (379 × 302 mm, LCD) and subtended 303 × 227 mm. The background was uniformly grey. The viewing distance was approximately 80 cm, although the participant's head position was not fixed by any apparatus. The image was presented until a response was made. Participants were told that there was no need to hurry to respond (i.e. self-paced task).

Each participant performed 64 experimental trials (32 objects in two views) for each task. In advance, a practice session (6 trials) was conducted in which the 6 practice objects were shown either in frontal view (3 trials) or in 3/4 view (3 trials). At the end of the experiment, any participant who performed the prediction task was debriefed that the cover story was fictional.

This experiment, as well as the subsequent experiments, was approved by the institutional review board and conducted in accordance with the Code of Ethics and Conduct (2009) of the Japanese Psychological Association and the Declaration of Helsinki. Written informed consent was obtained from all participants in advance.

#### 2.1.3 Participants

Forty-eight individuals were recruited and paid for their participation. Written informed consent was obtained in advance. They were graduate/undergraduate students of universities in/nearby Tokyo. As 8 had participated in other experiments using an identical set of stimuli, we omitted their data and analysed the remaining 40 (21 females, 19 males), who had an average age of 21.4 years (range 18–31).

Twenty participants (randomly chosen) performed both the likability rating task and the prediction task. In order to counterbalance any carryover effects between the tasks, we randomly divided participants into a rating-first group (*n* = 10), who performed the rating task first, and the prediction-first group (*n* = 10), who performed the prediction task first. The remaining 20 participants performed only either the rating task (*n* = 10, rating-only group) or the prediction task (*n* = 10, prediction-only group). Mean age was not significantly different among the four groups. Gender was as equalized as possible among the groups.

#### 2.1.4 Within- and between-group designs

We planned two designs of analysis, within- and between-group designs. In the within-group design, we examined the correlation between likability rating and prediction made by the identical set of participants, namely, the 20 who performed both tasks. In the between-group design, we examined a correlation between likability rating and prediction made by the different sets of participants. For this design, we adopted ratings made by the rating-only group and the rating-first group (*n* = 20 raters in total), and adopted predictions made by the prediction-only group and the prediction-first group (*n* = 20 predictors in total).

The 20 participants (11 females, 9 males) who performed both tasks had a mean age of 21.4 (range 18–31). The 20 raters (10 males, 10 females) in the between-group analysis had a mean age 20.6 (range 18–26). Finally, the 20 predictors (11 females, 9 males) in the between-group analysis had a mean age 22.1 (range 19–31).

### 2.2 Results

#### 2.2.1 Group analysis

First, we examined how well a group of 20 participants could predict the average likability rating of 20 participants. For each view of each object, the rated/predicted likability scores were averaged across participants. We examined the object-wise correlation between mean prediction and mean rating, which reflected prediction validity as a group. In the within-group analysis, the mean prediction was positively correlated with the mean rating, *r* = .85 (*t*(30) = 8.81, *p* < .001) for frontal view and *r* = .87 (*t*(30) = 9.88, *p* < .001) for 3/4 view. The correlations were also significant for the between-group analysis, *r* = .80 (*t*(30) = 7.41, *p* < .001) for frontal view and *r* = .68 (*t*(30) = 4.94, *p* < .001) for 3/4 view. As a group of 20 individuals, they successfully predicted the average liking of others.

#### 2.2.2 Individual analysis

**Figure 2.**
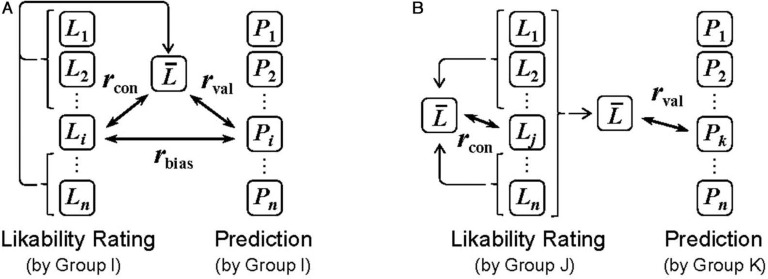
Schematic diagram of the three correlation coefficient indices (*r*_val_, *r*_con_, *r*_bias_) used in individual analysis. Each square represents a set of 32 values (rating/prediction for 32 stimulus objects). A Within-group design (each participant performed both the rating task and the prediction task), B between-group design (the two tasks were performed by separate groups). *L*_i_ and *P*_i_ represent likability ratings and predictions, respectively, made by participant *i*. $\bar{L}$ represents mean rating of others. It was either an average of *n* − 1 ratings or *n* ratings (depending on design and analysis). Prediction validity (*r*_val_) of participant *i* was defined as a correlation between *P*_i_ and $\bar{L}$. Rating consistency *r*_*con*_ was a correlation between *L*_i_ and $\bar{L}$. Prediction bias (*r*_bias_) was a correlation between *P*_*i*_ and *L*_*i*_, which was available only in the within-group analysis.

The central interest of the present paper was the validity of predictions made by individuals. To address this issue, for each participant, we computed three indices (Figure 2): prediction validity (*r*_val_), rating consistency (*r*_*con*_) and prediction bias (*r*_*bias*_).

Prediction validity *r*_val_ was defined as the correlation between a single participant's prediction (*P*_i_) and the mean of other participants' ratings $\bar{L}$. In the within-group design (Figure 2A), participant (*i*) was not included in $\bar{L}$, that is, $\bar{L}$ was the mean of the other 19 participants' ratings. This procedure prevented overestimation of *r*_val_. In the between-group design (Figure 2B), $\bar{L}$ was simply defined as the mean of ratings made by the 20 raters. Rating consistency *r*_con_ was the correlation between a single participant's rating (*L*_i_ or *L*_j_) and the mean of other participants' ratings (*L*). In both within- and between-group designs, the participant in question (*i* or *j*) was not included in $\bar{L}$ (i.e. $\bar{L}$ was the mean of the other 19 participants). Prediction bias *r*_bias_ was a correlation between a single participant's rating (*L*_*i*_) and the prediction by that same participant (*P*_*i*_). This index was available only in the within-group design.

The results of the three indices, averaged across participants, are listed in Table 1. Prediction validity *r*_val_ was approximately .3 on average for both analysis designs and both object views, which was lower than the prediction validity observed in the group analysis (.68–.87). This was not surprising, as individual differences in the data were overlooked by averaging in the group analysis.

**Table 1.** Results of individual analysis in Experiments 1 and 2. *r*_val_, prediction validity; *r*_con_, rating consistency; *r*_bias_, prediction bias. These indices were determined for each participant. This table shows their averages.

Stimulus	*r*_val_	*r*_con_	*r*_bias_
Experiment 1(within-group)			
Frontal view	.287	.303	.479
3/4 view	.324	.339	.509
Experiment 1 (between-group)			
Frontal view	.294	.360	–
3/4 view	.285	.306	–
Experiment 2 (within-group)			
Car, 3/4 view	.429	.381	.501
Chair, 3/4 view	.343	.338	.543

There were two critical findings here. First, *r*_val_ was not higher than *r*_con_; the mean likability rating $\bar{L}$, which was predicted by each individual participant, was equally correlated with individual prediction *P*_*i*_ and individual rating *L*_*i*_. In other words, participants could predict mean others' liking only to the degree to which the individual's own liking was similar to the mean others' liking. A repeated-measures analysis of variance (ANOVA) on Fischer's *Z*-transformed correlation coefficients (*r*_val_ and *r*_con_) confirmed this observation. For the within-group design, an ANOVA with two within-participant factors (2 indices × 2 views) showed no significant effect (main effect of index, *F* < 1; main effect of view, *F*(1,19) = 2.60, *p* = .12; interaction, *F* < 1). For the between-group design, a mixed-design ANOVA with two factors (index as between-participant factor and view as within-participant factor) found no significant effect (main effect of index, *F* < 1; main effect of view, *F*(1,38) = 1.38, *p* = .248; interaction, *F* < 1).

Second, *r*_bias_ was higher than *r*_val_; individual prediction *P*_i_ was more correlated with one's own rating *L*_i_ than the mean others' rating $\bar{L}$, even though the participants were required to predict $\bar{L}$. In order to confirm this observation, a repeated-measures ANOVA with two factors (2 indices × 2 views) was conducted on Fischer's *Z*-transformed correlation coefficients (*r*_val_ and *r*_bias_). This analysis was available only for the within-group design. The main effect of index was significant, *F*(1,19) = 19.35, *p* < .001, confirming that *r*_bias_ was higher than *r*_val_. Neither the main effect of view (*F* < 1) nor the interaction (*F* < 1) was significant.

#### 2.2.3 Analysis of consensus

We also conducted an analysis in the manner usually adopted in FCE studies—testing whether predicted consensus is higher than real consensus or not. This analysis was available only in the within-group design. First, we transformed the rating/prediction responses (1–7) to binary data by considering responses 1–3 as “bad” and responses 5–7 as “good”. The neutral response (4) was omitted. For each participant and each view of each object, we computed predicted consensus and real consensus. Predicted consensus is an agreement between prediction and rating made by the same participant. For instance, if participant *i* rated a chair as “good” and predicted others' ratings for the same chair as “good (bad)”, the predicted consensus for the object is 1 (0). We averaged this across objects. Real consensus is the proportion of others whose rating agreed with the participant's rating. For instance, if a chair was rated by participant *i* as “good”, and 12 of 19 other participants rated the chair as “good”, the real consensus was .63 (12/19). We averaged this across objects.

The mean predicted consensus and mean real consensus are shown in Table 2. For both views, predicted consensus was higher than real consensus. The two measurements were arcsine transformed and tested by a repeated-measures ANOVA with two factors (2 measurements × 2 views). The main effect of measurement was significant (*F*(1,19) = 61.83, *p* < .001), whereas the main effect of view (*F* < 1) and the interaction (*F* < 1) were not significant. These results confirmed the occurrence of FCE and were consistent with the finding that prediction bias *r*_bias_ was higher than prediction validity *r*_val_.

**Table 2.** Results of consensus analysis in Experiments 1 and 2.

Stimulus	Real consensus	Predicted consensus
Experiment 1 (within-group)		
Frontal view	.547	.758
3/4 view	.577	.758
Experiment 2 (within-group)		
Car, 3/4 view	.601	.796
Chair, 3/4 view	.585	.801

### 2.3 Discussion

The results clearly demonstrated that participants were not good at predicting others' average liking ratings. As *r*_val_ was not higher than *r*_con_, we did not find evidence that individual participants successfully utilized any knowledge on the difference between one's own liking and others' average liking. The result implies that, if one wants to predict others' liking of visual objects, there is no need to “predict”. Simply trusting one's own liking would be sufficient.

Did our participants give up on the prediction task and simply respond in the same manner as in the rating task? This did not seem to be the case. An additional analysis on response time revealed that participants spent a significantly longer time on the prediction task than they did on the rating task. We analysed log response times after excluding outlier response times (defined as shorter than 200 ms or longer than 10 s). As shown in the Additional Material, the mean log response time was longer for the prediction task in both views and both designs. An ANOVA demonstrated that the main effect of task was significant, *p* < .01. In addition, the grand averages of rating/prediction differed between the tasks (see Additional Material). The mean predicted likability score was significantly (*p* < .001) higher than the mean rated likability score in both views and both designs. In short, participants performed the two tasks differently. They tried to predict mean others' liking, but the results were not better than the correlation between one's own liking and others' liking.

The higher grand average for scores in the prediction task than in the rating task implies that participants predicted that others would like the objects more than they did. It may be more precise to say that the participants could not have correct knowledge of what others dislike, rather than what others like.

The second critical finding was that participants predicted others' liking to be more similar to their own liking than it really was. The analysis of consensus further supported this conclusion, namely, that the FCE occurred. These results imply that people do not consciously know the public favour of visual stimuli and discriminate it from one's own liking. Consequently, it seems plausible that a person with ordinary, similar-to-average liking would perform the prediction task well. In fact, for the 20 individuals who performed both tasks, their rating consistency *r*_con_ was positively correlated with prediction validity *r*_val_ (*r* = .57, *t*(18) = 2.95, *p* = .009 for frontal view; *r* = .75, *t*(18) = 4.76, *p* < .001 for 3/4 view). A similar finding was reported for predictions of a familiar individual's liking (Lerouge & Warlop, 2006).

## 3 Experiment 2

The stimulus objects used in Experiment 1 included various categories. However, in our daily lives, it seems more critical to predict others' like/dislike in a single object category, for instance, “this car will be liked by many others, but that car will not be liked”. Stich, Knäuper, Eisermann, and Leder (2007) showed that observers utilize different sets of aesthetic criteria for different object classes. Thus, if the stimulus objects are derived from a single category (e.g. car), observers might adopt a fixed set of evaluation criteria. Such a situation would increase prediction validity. Therefore, in Experiment 2, we focused on two sets of single-category object, namely, cars and chairs. In order to verify the robustness of result, we examined these two categories.

### 3.1 Method

#### 3.1.1 Participants

Twenty-one individuals were recruited and paid for their participation. Written informed consent was obtained in advance. Participants were graduate/undergraduate students of universities in/nearby Tokyo. For one participant, the experiment was terminated halfway through due to technical difficulties with the apparatus. We analysed the data of the remaining 20 participants (11 males, 9 females), who had a mean age of 21.9 years (range 19–35). None had participated in Experiment 1.

#### 3.1.2 Stimuli

We adopted 32 cars and 32 chairs as stimulus objects (see Additional Material). In addition, four cars and four chairs were used for practice trials. The objects were selected from available commercial packages of 3D object model data. The objects were rendered into coloured stimulus images in the same way as in Experiment 1. As FCE occurred equally for frontal views and 3/4 views in Experiment 1, we used 3/4 view only in Experiment 2 (Figure 1).

#### 3.1.3 Design and procedure

The apparatus was identical to that used in Experiment 1. The instructions to participants were also identical to those in Experiment 1, except that the stimulus images would show various examples of a single category (car/chair). Each participant performed both rating and prediction tasks, and the order of the tasks was counterbalanced. In each task, two sessions were conducted, namely, a car session and a chair session. Each session had 4 practice trials and 32 experimental trials. The order of object category was also counterbalanced; half of the participants performed the car session first, while the other half performed the chair session first. Thus, each participant performed 4 sessions (2 tasks × 2 object categories). The mean age of participants was not significantly different among the four groups (rating/prediction first × car/chair first). Gender was as equalized as much as possible among the groups. A self-paced break was given in between the sessions. At the end of the experiment, participants were debriefed that the cover story for the prediction task was fictional.

### 3.2 Results

#### 3.2.1 Group analysis

The mean prediction of the 20 participants was highly correlated with the mean rating, *r* = .88, *t*(30) = 10.40, *p* < .001 for the car stimuli and *r* = .84, *t*(30) = 8.38, *p* < .001 for the chair stimuli. As in Experiment 1, the group of 20 predicted the mean liking well.

#### 3.2.2 Individual analysis

In the same manner as in Experiment 1, we examined individual prediction validity (*r*_val_), rating consistency (*r*_con_) and prediction bias (*r*_bias_) for each object category. Only the within-group design (Figure 2A) was adopted in Experiment 2. A summary of the indices is shown in Table 1. Prediction validity *r*_val_ was not higher than rating consistency *r*_con_. A repeated-measures ANOVA (2 indices × 2 object categories) on the Fischer's *Z*-transformed *r*_val_ and *r*_con_ found no significant effect (*F* < 1 for main effects and interaction). Prediction bias *r*_bias_ was higher than prediction validity *r*_val_, and the ANOVA on the Fischer's *Z*-transformed *r*_bias_ and *r*_val_ revealed a significant main effect of index (*F*(1,19) = 15.74, *p* < .001), while the main effect of object category and the interaction were not significant (*F* < 1 and *F*(1,19) = 1.96, *p* = .178, respectively). In summary, the pattern of results was qualitatively similar to that of Experiment 1. There were no reliable differences among the object categories.

#### 3.2.3 Analysis of consensus

We compared predicted consensus and real consensus in the same way as in Experiment 1 (Table 2). For both object categories, predicted consensus was higher than real consensus; according to an ANOVA (2 measurements × 2 object categories) on arcsine-transformed predicted/real consensus measurements, the main effect of measurement was significant (*F*(1,19) = 86.02, *p* < .001). Neither the main effect of object category (*F* < 1) nor the interaction (*F* < 1) was significant. As in Experiment 1, FCE was observed.

### 3.3 Discussion

We replicated the pattern of results from Experiment 1. The failure in predicting others' liking was observed again for the likability ratings of single-category objects (car/chair). We also confirmed that mean log RT was longer for the prediction task than the rating task (see Additional Material), suggesting that the participants did not give up on the prediction task, but the predictions were biased towards one's own liking.

## 4 Experiment 3

In the preceding experiments, the fictional age/gender profile in the cover story for the prediction task was comparable to the actual profile of the participants themselves. Such a situation might encourage the participants to utilize their own liking as reference data. If so, it is reasonable that they drew their predictions to their own liking, resulting in the biased prediction. Indeed, some studies showed that FCE is dependent on attributes of the group for which the liking is predicted (Bosveld et al., 1995; Hoch, 1987). In Experiment 3, therefore, we asked participants to predict the mean liking of a group that was different from the participants, namely, the other-gender group.

### 4.1 Method

The stimuli were identical to those used in Experiment 1. Forty participants (20 males, 20 females) participated in the experiment. The mean age was 23.0 (range 21–29) and 22.7 (range 19–26) for male and female participants, respectively. They were graduate/undergraduate students of universities in/nearby Tokyo. All the participants performed both rating and prediction tasks. The order of the tasks was roughly counterbalanced; for each gender group, 11 participants performed the rating task first, and the remaining 9 performed the prediction task first. Age and gender were as equalized as much as possible among the sub-groups of task order.

The instructions for the rating task were identical to those in Experiment 1. In the prediction task, the participants were given a fictional cover story, which stated that 40 individuals (20 men, 20 women) living in Tokyo had performed the rating task. Then, the male (female) participants were asked to “predict the result of the 20 women (men)”, namely, the average of the likability ratings made by the 20 individuals. As a part of the cover story, participants were also told that the 20 women (men) were 21.0 years of age on average, ranging from 18 to 32. In addition, the procedure of the rating task was explained to the participants.

### 4.2 Results

#### 4.2.1 Group analysis

Overall, the mean prediction was positively correlated with the mean rating. The mean prediction by the 20 male participants was correlated with the mean rating by the 20 female participants, *r* = .61, *t*(30) = 4.19, *p* < .001 for frontal view and *r* = .57, *t*(30) = 3.84, *p* < .001 for 3/4 view. The mean prediction by the 20 female participants was also correlated with the mean rating by the 20 male participants, *r* = .64, *t*(30) = 4.55, *p* < .001 for frontal view and *r* = .40, *t*(30) = 2.37, *p* = .025 for 3/4 view. Again, as a group, both gender groups well predicted the mean liking of the other-gender group. The correlation between the mean rating by males and the mean rating by females was *r* = .55 for frontal view (*t*(30) = 4.19, *p* = .001) and *r* = .57 for 3/4 view (*t*(30) = 3.80, *p* < .001).

#### 4.2.2 Individual analysis

In the same way as in the preceding experiments, *r*_val_, *r*_con_ and *r*_bias_ were calculated for each participant. Note that *r*_val_ /*r*_con_ here indicates a correlation between individual prediction/rating and mean rating of the other-gender group, not the same-gender group (Table 3).

**Table 3.** Results of individual analysis in Experiment 3. *r*_val_, prediction validity; *r*_con_, rating consistency; *r*_bias_, prediction bias. These indices were determined for each participant. This table shows their averages.

Stimulus	*r*_val_	*r*_con_	*r*_bias_
Male participants			
Frontal view	.267	.209	.501
3/4 view	.259	.233	.454
Female participants			
Frontal view	.282	.232	.399
3/4 view	.175	.206	.332

First, we compared prediction validity *r*_val_ with rating consistency *r*_con_. On the Fischer's *Z* transforms of *r*_val_ and *r*_con_, we conducted a mixed-design ANOVA with three factors—gender as a between-group factor and index (*r*_val_/*r*_con_) and view (frontal/three-quarter) as within-group factors. No main effect was significant, with *F* < 1, *F*(1,38) = 1.84, *p* = .183, and *F*(1,38) = 1.46, *p* = .235, for gender, index and view, respectively. Interactions were also non-significant (*p* > .1). In both gender groups, *r*_val_ was not higher than *r*_con_. Next, we compared prediction validity *r*_val_ with prediction bias *r*_bias_. On the Fischer's *Z* transforms of the indices, a 3-way factorial ANOVA (gender × index × view) was conducted. The main effect of gender was not significant (*F*(1,38) = 1.76, *p* = .193), whereas main effects of index and object view were significant, *F*(1,38) = 21.63, *p* < .001 and *F*(1,38) = 5.07, *p* = .030, respectively. No interaction was significant (*p* > .1). Consistent with Experiments 1 and 2, in both gender groups, *r*_bias_ was higher than *r*_val_.

#### 4.2.3 Analysis of consensus

Predicted consensus and real consensus were examined (Table 4). A mixed-design ANOVA with gender as a between-group factor and measurement (predicted/real consensus) and view (frontal/three-quarter) as within-group factors was conducted on arcsine-transformed predicted/real consensus data. The main effect of gender showed a trend toward significance, *F*(1,38) = 3.83, *p* = .058. The main effect of measurement was significant (*F*(1,38) = 67.04, *p* < .001) and the interaction with gender was significant (*F*(1,38) = 7.30, *p* = .010). As reflected in this interaction, the simple main effect of gender was significant for predicted consensus (*F*(1,76) = 10.20, *p* = .002) but not for real consensus (*F* < 1). In other words, male participants' predicted consensus was higher than female participants' predicted consensus, although there was no gender difference in real consensus. The simple main effect of measurement was significant in both gender groups (*p* < .001), confirming that FCE occurred irrespective of gender. Additionally, the main effect of view was significant (*F*(1,38) = 5.07, *p* = .030). All other effects were non-significant.

**Table 4.** Results of consensus analysis in Experiment 3.

Stimulus	Real consensus	Predicted consensus
Male participants		
Frontal view	.550	.825
3/4 view	.600	.831
Female participants		
Frontal view	.544	.710
3/4 view	.613	.725

### 4.3 Discussion

Experiment 3 demonstrated that the prediction of mean liking of the other gender is similarly unreliable. Consequently, it was implied that the biased predictions in Experiments 1 and 2 were not due to the experimental design in which the participants were asked to predict liking of people similar to themselves. Analysis on consensus revealed the reliable FCE as well.

One curious finding was the gender difference in FCE; predicted consensus was higher for the male participants than it was for the female participants. Therefore, we further examined FCE size, defined as predicted consensus minus real consensus. An ANOVA (gender × view) revealed that FCE size was significantly larger for the male participants than for the female participants (*F*(1,19) = 5.69, *p* = .028). Although FCE occurred in both gender groups, the magnitudes of the effect were not equal. Similarly, mean *r*_bias_ tended to be higher for male participants than for female participants (Table 3), though this difference was not statistically significant.

## 5 General discussion

If people consciously know the public favour of visual stimuli and discriminate it from their own liking, they could predict others' liking better than they could by simply relying on their own liking. The results of the present study showed this is not the case; the validity of prediction was not as good as the consistency of one's own liking with the public favour. Further, the predictions were biased towards one's own liking. FCE was replicated as well, irrespective of multiple-category versus single-category objects.

These findings suggest that people do not have correct knowledge of the difference between their own liking and public favour. Because unconscious, automatic processes/factors play a considerable role in preference judgments, it would be more difficult to understand one's own liking than we believe. In addition, it is also likely that prediction of an individual other's liking (e.g. friend, neighbour) is more critical for determining our social behaviour, and our mind is tuned more to individual liking than to public favour. The current finding may provide new insights into the adaptive function of favour/preference, which is still far from fully understood.

### 5.1 Is group prediction biased as well?

As shown by the group analysis, the predictions as a group (i.e. mean prediction) were highly correlated with actual mean liking. On the other hand, the prediction validity analyses revealed that individual prediction was biased towards one's own liking. Is this also the case for prediction as a group? We addressed this issue with the data from Experiments 1 and 3. As predictors, we adopted 20 participants who performed both rating and prediction tasks in Experiment 1. As a target to be predicted, we adopted the mean rating by 22 participants (11 males, 11 females) who performed the rating task first in Experiment 3. The group *r*_val_, a correlation between mean prediction by the predictors and the target mean rating, was .642 and .681 for frontal and 3/4 view, respectively. The group *r*_bias_, a correlation between mean prediction by the predictors and the mean rating by the same predictors, was .849 and .875 for frontal and 3/4 view, respectively. Therefore, it seems that group *r*_bias_ was higher than group *r*_val_, confirming that prediction as a group is also biased towards the group's mean liking.

### 5.2 The power of number of predictors

**Figure 3.**
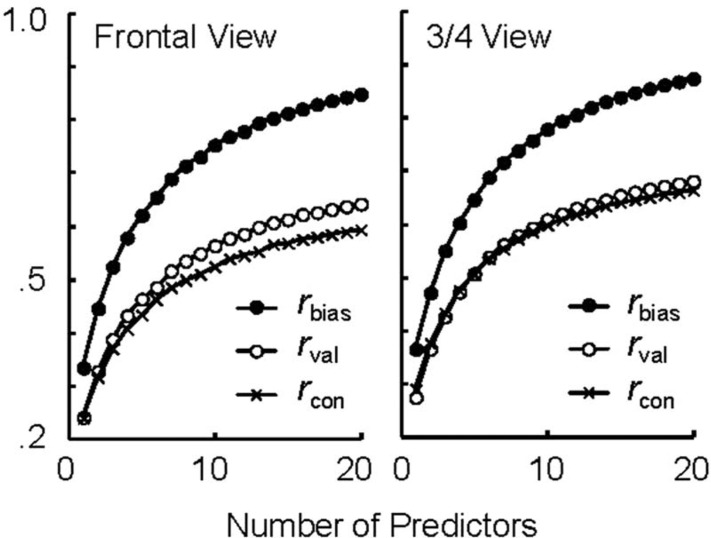
Prediction bias (*r*_bias_), prediction validity (*r*_val_) and rating consistency (*r*_con_) as a function of number of people who predicted the mean likability rating. This analysis is based on the data of Experiments 1 and 3. *r*_bias_ is a correlation between the mean prediction by predictors and the mean likability rating of identical predictors. *r*_val_ is a correlation between the mean prediction and the actual mean rating by the target group (22 individuals from Experiment 3). *r*_con_ is a correlation between the mean rating of the predictors and the mean rating by the target group (22).

It is noteworthy that increasing the number of predictors would yield greater prediction validity. As noted earlier, the group *r*_val_ was .642 and .681, which seems much higher than mean individual *r*_val_ observed in Experiments 1 and 3. This was likely because individual variations in prediction were compromised by averaging across predictors. We conducted a simulation in which the effect of predictor number was examined. Given the 20 predictors in Experiment 1 and the target mean rating by 22 from Experiment 3 (as noted earlier), we computed mean *r*_val_, *r*_con_ and *r*_bias_ for each number of predictors (*n* = 1–20). For instance, when *n* = 5, we randomly selected 5 individuals among the 20 predictors, and *r*_val_ was computed as a correlation between mean prediction by the 5 and the target mean rating by the 22. We tried a maximum of 1,000 combinations of 5 predictors and averaged the results. The case of *n* = 1 corresponds to individual *r*_val_, *r*_con_ and *r*_bias_, and the case of *n* = 20 corresponds to the group *r*_val_, *r*_con_ and *r*_bias_ described earlier. Figure 3 shows the result. As discussed earlier, the predictions were biased towards own liking values, irrespective of *n*, but the absolute value of prediction validity increases drastically as a function of *n*. That is, the prediction validity is simply a function of the number of predictors.

### 5.3 Remaining issues

Interestingly, we found a gender difference in FCE. Experiment 3 showed that the magnitude of FCE was greater for male participants than it was for female participants. There was no significant gender difference in FCE size in any other experiment (*p* > .1). Hence, the more biased prediction by male participants was found only when the task was to predict the liking of the other-gender (i.e. female) group. In other words, male participants tended to judge that the female group would similarly like particular visual objects. An analogous phenomenon was reported by Marcus and Miller (2003), who showed that women know face attractiveness as rated by other-gender individuals better than men do. However, the cross-gender effect warrants further investigation.

Another remaining issue is the effect of object class. We tested everyday objects in this study, and it is still unclear whether the current finding holds for other object classes (e.g. faces, artwork), as aesthetic criteria can differ across different object classes (Stich et al., 2007) and/or there may be less consensus in aesthetic criteria for everyday objects than those for faces and artwork.

### 5.4 Conclusions

In conclusion, our participants showed little indication that prediction was better than making decisions based on their own rating when judging others' public favour of visual objects. That is, people do not seem to have access to or utilize knowledge of public favour. Thus, the lesson from the current study is simple, namely, do not make predictions independently (because it is not better than choosing what you like), but rather, ask other people, as two heads are better than one.
